# Dielectric Manipulated Charge Dynamics in Contact Electrification

**DOI:** 10.34133/2022/9862980

**Published:** 2022-02-01

**Authors:** Kunming Shi, Bin Chai, Haiyang Zou, Daomin Min, Shengtao Li, Pingkai Jiang, Xingyi Huang

**Affiliations:** ^1^Department of Polymer Science and Engineering, Shanghai Key Laboratory of Electrical Insulation and Thermal Aging, State Key Laboratory of Metal Matrix Composites, Shanghai Jiao Tong University, Shanghai 200240, China; ^2^School of Materials Science and Engineering, Georgia Institute of Technology, Atlanta, GA 30332-0245, USA; ^3^State Key Laboratory of Electrical Insulation and Power Equipment, Xi'an Jiaotong University, Xi'an, Shaanxi 710049, China

## Abstract

Surface charge density has been demonstrated to be significantly impacted by the dielectric properties of tribomaterials. However, the ambiguous physical mechanism of dielectric manipulated charge behavior still restricts the construction of high-performance tribomaterials. Here, using the atomic force microscopy and Kelvin probe force microscopy, an in situ method was conducted to investigate the contact electrification and charge dynamics on a typical tribomaterial (i.e., BaTiO_3_/PVDF-TrFE nanocomposite) at nanoscale. Combined with the characterization of triboelectric device at macroscale, it is found that the number of transferred electrons increases with contact force/area and tends to reach saturation under increased friction cycles. The incorporated high permittivity BaTiO_3_ nanoparticles enhance the capacitance and electron trapping capability of the nanocomposites, efficiently inhibiting the lateral diffusion of electrons and improving the output performance of the triboelectric devices. Exponential decay of the surface potential is observed over monitoring time for all dielectric samples. At high BaTiO_3_ loadings, more electrons can drift into the bulk and combine with the induced charges on the back electrode, forming a large leakage current and accordingly accelerating the electron dissipation. Hence, the charge trapping/storing and dissipating, as well as the charge attracting properties, should be comprehensively considered in the design of high-performance tribomaterials.

## 1. Introduction

Contact electrification brings about charge transfer between two materials during contact or friction process [1–3], and this universally existing phenomenon is also considered to be derived from the difference of work functions or surface potentials between the contacting materials [4–6]. Though the triboelectric charges are commonly regarded to be detrimental for electronic circuits and systems, they have been successfully utilized in today's advanced technologies, including photocopying [7], laser printing [8], electrostatic separation [9], painting [10], field-effect transistor [11–13], and mechanical energy harvesting [14–16].

In the field of energy harvesting, triboelectric devices, a new sustainable power source based on contact electrification and electrostatic induction effect, can convert ambient mechanical energy to electrical energy [17–21]. The triboelectric charges on material surface serve as an electrostatic induction source to generate electricity, and the output voltage and current are both dictated by surface charge density [22, 23]. Hence, boosting the amount of triboelectric charge during contact electrification is essential for the improvement of triboelectric device performance. The dielectric properties of the tribomaterials play a key role in triboelectric performance [24–26]. Organic dielectrics-based devices exhibit a limited output performance owing to their relatively low permittivity. Incorporating high permittivity (high-*k*) fillers into the polymer matrix has been demonstrated to be efficient to improve the surface charge density and triboelectric performance [27–31]. Though this strategy has been well established in the construction of tribomaterials, the physical interpretation of dielectric manipulated charge dynamics (charge transfer, charge distribution, and charge dissipation) still remain ambiguous, which restricts the further optimization of tribomaterials. To figure out the mechanism of dielectric manipulation, quantitative investigation of the in situ contact electrification and charge dynamics on the tribomaterials surface at nanoscale is required, as the regular macroscopic experiments cannot accurately control the electrification process or directly reveal the electrification interface [32].

Atomic force microscopy (AFM) is a high-precision instrument, which can characterize a variety of material properties at nanoscale for multifarious applications [33]. Due to the advantage of precise control of contact force, area, and friction cycle of the tip on the sample, contact electrification can be achieved and accurately controlled in the contact mode of AFM [34, 35]. Furthermore, with a high resolution, the Kelvin probe force microscopy (KPFM) can detect the surface potential in real time, which can be utilized to interpret surface charges [36, 37]. Thus, the charge transfer, charge distribution and the subsequent charge dissipation processes on the dielectric surface can be systematically investigated via the combination of AFM and KPFM techniques.

Herein, contact electrification and charge dynamics at nanoscale are in situ and quantitatively investigated for a metal-dielectric case using AFM and KPFM, as a function of barium titanate nanoparticles (BTO NPs) density inside poly(vinylidene fluoride-co-trifluoro-ethylene) (PVDF-TrFE). In the nanocomposites, PVDF-TrFE has tremendous charge-attracting and transfer properties, while high-*k* BTO nanoparticle owns a high internal polarization and excellent charge trapping capability. Mechanical conditions of contact force and friction cycle are studied on the spin coating PVDF-TrFE sample as they can significantly affect the charge transfer during contact electrification. Particularly, BTO/PVDF-TrFE nanocomposites with different dielectric properties are fabricated and characterized to demonstrate the dielectric manipulation on the charge transfer, distribution, and dissipation processes. The triboelectric performance of the macroscopic devices was also investigated to obtain a comprehensive analysis and understanding. A proposed mechanism is finally exhibited to elucidate the dielectric manipulated charge dynamics and surface charge density.

## 2. Results and Discussion

**Figure 1:**
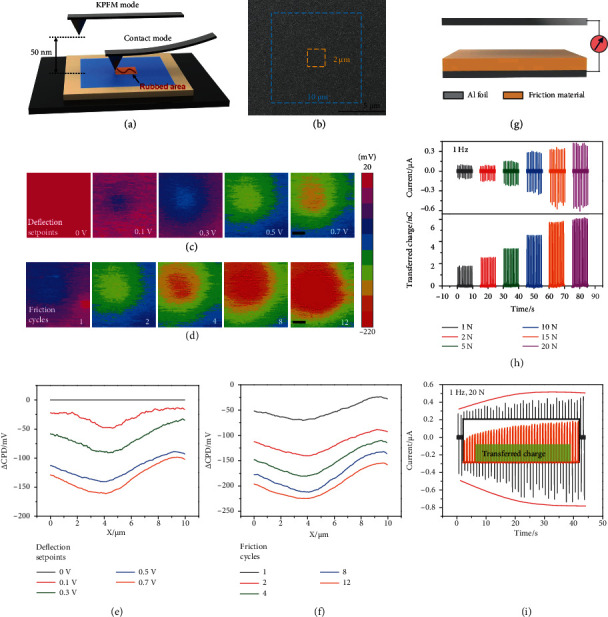
(a) Schematic illustration of the experiment at nanoscale. (b) SEM of PVDF-TrFE surface and the demonstration of different areas for friction and potential characterization. (c, d) *Δ*CPD distribution and (e, f) the corresponding cross-section profiles detected under different deflection setpoints (friction cycle: 2) and number of friction cycles (deflection setpoint: 0.5 V). The scale bar is 2 *μ*m. (g) Structure of a triboelectric device. (h) Output current and transferred charges of the device operating under different contact forces. (i) Output current as a function of measuring time; the inside shows the variation of transferred charge.

Figure 1(a) illustrates the experimental procedure via AFM and KPFM techniques. Briefly, the sample was primarily scanned by KPFM for a pristine surface potential; subsequently, the contact mode of AFM was switched for an in situ contact electrification in a relatively small area. Finally, the surface potential of the sample was immediately detected again by KPFM at the initial large size. Figure 1(b) shows the surface morphology of PVDF-TrFE sample and the characterized area of surface potential (10 × 10 *μ*m^2^) and contact electrification (2 × 2 *μ*m^2^). Basically, surface potential (i.e., contact potential difference (CPD)) of the sample was determined by two components: work function of the sample and electrostatic charges on the sample [32]. To exclude the different surface properties of the samples, the surface potential after contact electrification was subtracted by the one measured before contact electrification. Thus, the difference value, termed as *Δ*CPD in this work, can directly demonstrate the influence of contact electrification on the surface potential.

As contact electrification can be affected by mechanical conditions [38, 39], Figure 1(c) shows the *Δ*CPD of PVDF-TrFE sample under different contact forces (directly proportional to the deflection setpoint, detail calculation shown in experimental section). It can be seen that the sample was negatively charged during contact electrification, indicating the electron transfer from tip to sample due to the electron transfer dominated mechanism in the metal/dielectric pairs [40–43]. It is worth noting that the negatively charged area of the sample is larger than the rubbed area; this phenomenon can be ascribed to the fact that the electrons diffuse from the rubbed area to adjacent area through the plane, radially [44]. Despite the electron diffusion, the sample demonstrates the highest *Δ*CPD in the rubbed area after contact electrification, which can be also distinguished from the *Δ*CPD profiles in Figure 1(e). In addition, the *Δ*CPD of the sample, in either a rubbed area or adjacent area, is more negative under a higher deflection setpoint, and the peak *Δ*CPD increases with deflection setpoint (Figure S1a). This is because a larger contact force leads to a larger contact area between tip and sample at nanoscale, thus facilitating the transfer of more electrons to sample. The investigation of repeated charge injection was conducted by varying the number of friction cycle under the deflection setpoint of 0.5 V. On the sample, the distance between each rubbed area for a fixed friction cycle number (e.g., 1, 2, 4, 8, and 12) is larger than 100 *μ*m. As shown in Figures 1(d) and 1(f), the *Δ*CPD of PVDF-TrFE increases along with the number of friction cycle, suggesting that electrons are transferred to the sample at each friction cycle, and high charge density can be achieved by multi-friction. Particularly, the *Δ*CPD dominated by triboelectric charges increases at a slowing rate. The peak *Δ*CPD in Figure S1b reveals that the electrons accumulate fast at a low number of friction cycle and tend to reach saturation at a high number of friction cycle. The reduced increasing rate of *Δ*CPD can be understood that the accumulated electrons could change the “effective work function” of the sample and inhibit the injection of more electrons [32].

To further certify the influence of mechanical condition, the contact electrification at macroscale was conducted by assembling Al foil and dielectric PVDF-TrFE film into a triboelectric device, as displayed in Figure 1(g). The contact electrification of the metal/dielectric pair was controlled by a liner motor with a force sensor. Under the operating frequency of 1 Hz, both the output current and the calculated transferred charge shown in Figure 1(h) increase along with the contact force in the range of 1 N-20 N. Otherwise, the output current of the device (1 Hz, 20 N) is plotted in Figure 1(i) as a function of measuring time. With the increasement of measuring time, the corresponding current and transferred charge increase and reach to saturation, gradually. These results concluded from both microscopic and macroscopic measurements are consistent with each other.

**Figure 2:**
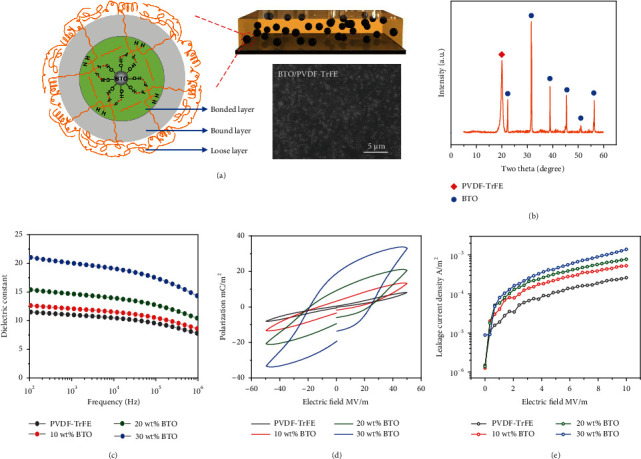
(a) Schematic illustration of the BTO/PVDF-TrFE nanocomposite and the hierarchical interface; the bottom shows the SEM morphology of the nanocomposite surface, (b) XRD pattern, (c) dielectric constant, (d) P-E loop, and (e) leakage current of BTO/PVDF-TrFE nanocomposites.

Dielectric properties of tribomaterials are crucial factors that affecting contact electrification [26, 45, 46]. To reveal the effect of dielectric properties on the charge dynamics, a series of nanocomposites based on BTO/PVDF-TrFE have been prepared.  Figure 2(a) schematically illustrates the BTO/PVDF-TrFE nanocomposite and the microinterface forming between BTO NPs and PVDF-TrFE matrix. The hierarchical interface consisting of bonded layer, bounded layer, and loose layer has tremendous impact on nanoparticle surface states, polymer chain configurations, inorganic/organic compatibility, and local interfacial electrical and dielectric behaviors [47–49]. The surface morphology of BTO/PVDF-TrFE nanocomposite shows the uniform dispersion of BTO NPs in PVDF-TrFE matrix, indicating their good compatibility. XRD in Figure 2(b) demonstrates both the characteristic peaks of BTO and PVDF-TrFE without any impurity and reveals the polar *β* phase-dominated crystallization in PVDF-TrFE [50].

Dielectric properties of the nanocomposites were investigated in range of 10^3^-10^6^ Hz at room temperature. As shown in Figure 2(c), the dielectric constant of PVDF-TrFE is 11.0 at 10^3^ Hz, while the dielectric constant of the nanocomposites increases with increasing BTO content and achieves a high value of 20.1 at 10^3^ Hz with a BTO content of 30 wt%. The dielectric loss of the nanocomposites is almost the same with PVDF-TrFE at high frequency, just a slight increase at low frequency (Figure S2). The increased dielectric constant of the nanocomposites relates to their enhanced capability of electric polarization, which facilitates the charge induction on the back electrode (schematically shown in Figure S3) [28].  Figure 2(d) shows that the polarization increases with electric field and BTO content. The 30 wt% BTO/PVDF-TrFE nanocomposite forms a 4.2 times stronger polarization (33.8 mC m^−2^) than that of PVDF-TrFE (8.1 mC m^−2^) under an electric field of 50 MV m^−1^. Otherwise, contact electrification consists of three subprocesses: charge generation, charge storage, and charge loss [51]. The dielectric constant is directly proportional to the capacitance and capability of the friction layer in storing charges, which is a determinant of surface charge density [51, 52], while, as a detrimental factor, leakage current forming across the friction layer could significantly decrease the surface charge density [28, 44, 53]. As displayed in Figure 2(e), the leakage current density of the nanocomposites increases along with BTO content under the electric field of 0-10 MV m^−1^, which may be caused by BTO NPs aggregation, interface overlapping, and the increasement of introduced defects.

**Figure 3:**
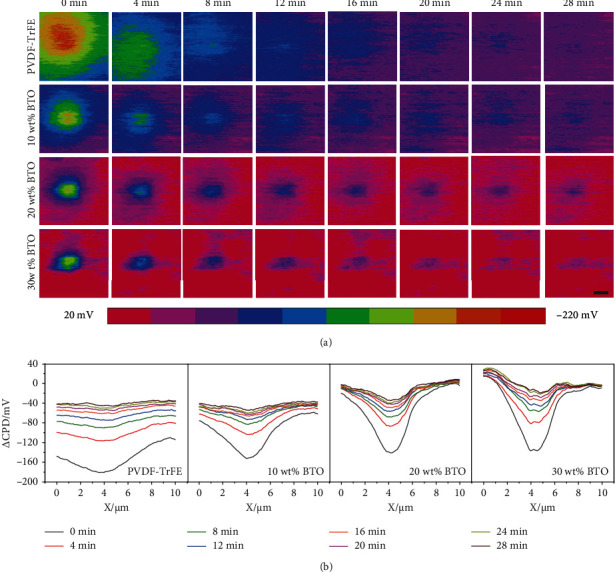
(a) *Δ*CPD distribution and (b) cross-section profiles of BTO/PVDF-TrFE nanocomposites at different time after contact electrification. Deflection setpoint: 0.5 V, number of friction cycle: 4. The scale bar is 2 *μ*m.

**Figure 4:**
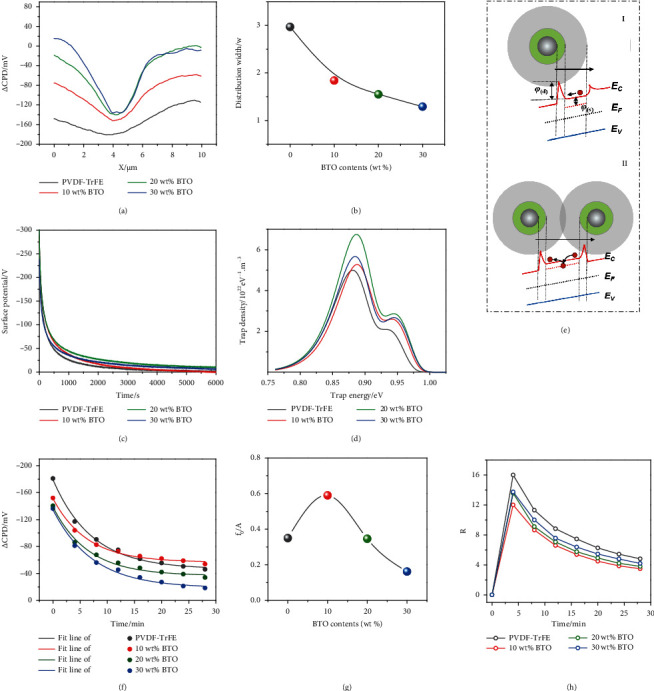
(a) *Δ*CPD profiles of BTO/PVDF-TrFE nanocomposites measured at 0 min and (b) the corresponding distribution width of Gaussian function fitting with *Δ*CPD profiles in (a). (c) Surface potential decay of the nanocomposites after being charged. (d) Trap density versus trap energy calculated by ISPD model. (e) Schematic illustration of the interfacial effect on local trap state. (f) Peak *Δ*CPD decay profiles of the nanocomposites along the monitoring time and the corresponding fitting lines. (g) The ratio of *f*_0_ to *A* for different composites. (h) The average decay rate of peak *Δ*CPD for different composites.

Contact electrification on dielectric manipulated nanocomposites was carried out using the in situ technique, and the charge dynamic was investigated via repeatedly recording the surface potential distribution of the nanocomposites every 4 min after contact electrification (deflection setpoint: 0.5 V, friction cycle: 4). As shown in Figure 3(a), *Δ*CPD of the nanocomposite varies with BTO content, and all decrease along with the monitoring time, which also can be seen from the *Δ*CPD distribution profiles extracted from the same cross-section of each image in Figure 3(b). Comparing the *Δ*CPD measured at 0 min of each nanocomposite, the negatively charged area becomes smaller with BTO content increasing from 0 wt% to 30 wt%. In Figure 4(a), one can see that the half width at a full maximum of the *Δ*CPD profile and the surface potential of adjacent area decrease along with BTO content. To quantitatively analyze the degree of charge diffusion on the sample surface, the *Δ*CPD profiles were fit with Gaussian distribution function *f*(*x*):

(1) $$\begin{array}{c} f\left(x\right)=f_{0}+C∙\exp\left[-\frac{{\left(x-x_{0}\right)}^{2}}{2w^{2}}\right]. \end{array}$$

In equation (1), *f*_0_ and *C* is the offset and magnitude constant for *f*(*x*), respectively, and *w* is the distribution width of the Gaussian function, which represents the degree of charge diffusion. In Figure 4(b), the value of *w* decreases from 3.0 to 1.3, even to 0.88 at a higher BTO content of 40 wt% (Figure S4). The result indicates that electron diffusion from rubbed area to adjacent area can be inhibited by the addition of BTO NPs, efficiently. It is ascribed to the fact that high-*k* BTO NPs can greatly enhance the interfacial polarization and capacitance of the nanocomposite, leading to a high capability to store/sustain the generated electrons at local surface [51]. Meanwhile, charge trapping sites in the nanocomposites can significantly influence the surface charge [54].

To evaluate the trap distributions in the nanocomposites, temporal distributions of surface potentials of the nanocomposites after being negatively charged were investigated, and the theoretical model of isothermal surface potential decay (ISPD) [55, 56] was applied to calculate charge trap density versus the charge trap energy. In Figure 4(c), the surface potential of all nanocomposites presents an exponential decay, which is due to the fact that the captured charges in the traps can escape via the thermionic-emission effect [57, 58] and then drift to the back electrode via a self-excited electric field. In addition, the injected charges in shallow traps can be easy to escape, thus leading to a rapid *Δ*CPD decay at early stage, while the injected charges in deep traps are difficult to escape, thus leading to a slow *Δ*CPD decay at last stage [55, 59]. The cross-over phenomenon of the decay curves is caused by the different initial surface potential and different charge decay rates of the samples [59]. Figure 4(d) shows the trap distribution of the nanocomposites analyzed via the model of ISDP. One can see that PVDF-TrFE exists two peaks at 0.88 eV and 0.94 eV, which corresponds to the energy level of shallow trap and deep trap in the semicrystalline polymer, respectively. After adding BTO NPs, both peaks move to a higher energy level, and the trap density increases along with BTO content, achieving a maximum value at a BTO content of 20 wt%. Upon the addition of BTO NPs, numerous matrix/nanoparticle interfaces with high potential barrier (especially for the bonded layer) are created in nanocomposites, which could efficiently trap the injected electrons and inhibit their migration via strong coulomb effect (Figures 4(e)-I), while excessive BTO NPs could lead to the overlapping of interfacial regions and the aggregation of BTO NPs, thus facilitating the charge migration in a longer rang and reducing the interfacial trap density (Figures 4(e)-II).

On the other hand, Figure 3 also shows that *Δ*CPD of all samples decay along with monitoring time, suggesting the charge dissipation occurs on the sample. Figure 4(f) depicts the peak *Δ*CPD of the nanocomposites decaying with monitoring time, exponentially. Generally, the charge dissipation can be ascribed to the following aspects [44]:

1. Ambient atmosphere and humidity
2. Charges diffusing laterally on the surface
3. Charges diffusing vertically into the bulk
4. Charges drifting to the electrode and combining with the induced charges on the electrode

At an ambient condition, charges can disperse into the atmosphere or combine with opposite charges from the atmosphere [44]. In addition, the humidity/moisture can create a water layer on the sample surface, which strongly increase the charge dissipation rate due to the screening of surface charges and improved surface conductivity for leakage [2, 4, 37, 44]. In terms of charge diffusion, the distribution width *w* of the nanocomposites in Figure S5 keeping almost unchanged over the monitoring time indicates the insignificant lateral diffusion of electrons in *Δ*CPD decay. The lateral diffusion of electrons mainly occurs at initial instant of charge injection, due to the high concentration gradient of electrons on sample surface. However, the accumulated electrons on contact surface can induce positive charges on the back electrode, and the established electric field could enhance the vertical diffusion of electrons into the bulk [60]. Furthermore, the electrons drifting deep could combine with the induced positive charges on the back electrode and form the leakage current across the film, thus significantly decreasing the electron density on the sample surface [28, 53]. Generally, the exponential decay curves in Figure 4(f) follow the equation below:

(2) $$\begin{array}{c} f\left(t\right)=A∙e^{-at}+f_{0}, \end{array}$$

where *A* denotes the attenuation component of *Δ*CPD, *f*_0_ is the residual steady-state component of *Δ*CPD resulting from the tightly bound charges, and *α* presents decay coefficient. It can be seen that the fitting lines are consistent with the measured data, and the ratio of *f*_0_ to *A* in Figure 4(g) tends to increase and then decrease with increasing BTO content, which means that the sample of 10 wt% BTO/PVDF-TrFE nanocomposite has a higher capability to retain electrons. Otherwise, the average decay rate of *Δ*CPD can be calculated by

(3) $$\begin{array}{c} R=\frac{{\Delta \text{CPD}}_{t}-{\Delta \text{CPD}}_{0}}{t}. \end{array}$$

In the equation, ΔCPD_0_ and ΔCPD_*t*_ are the ΔCPD measured at 0 min and *t* min, respectively. The result in Figure 4(h) shows that 10 wt% BTO/PVDF-TrFE sample has the lowest average decay rate during all the monitoring time. This is because the addition of BTO NPs can increase the dielectric capacitance and electron trapping ability of the sample, while excessive BTO NPs can lead to an increment of leakage current in the nanocomposite, as shown in Figure 2(e), which would cancel out the triboelectric charges on the tribomaterial surface and the induced charges on the back electrode. Therefore, the two antagonistic impacts of adding high-*k* BTO NPs codetermine the surface charge density, thus leading to an optimal BTO content.

**Figure 5:**
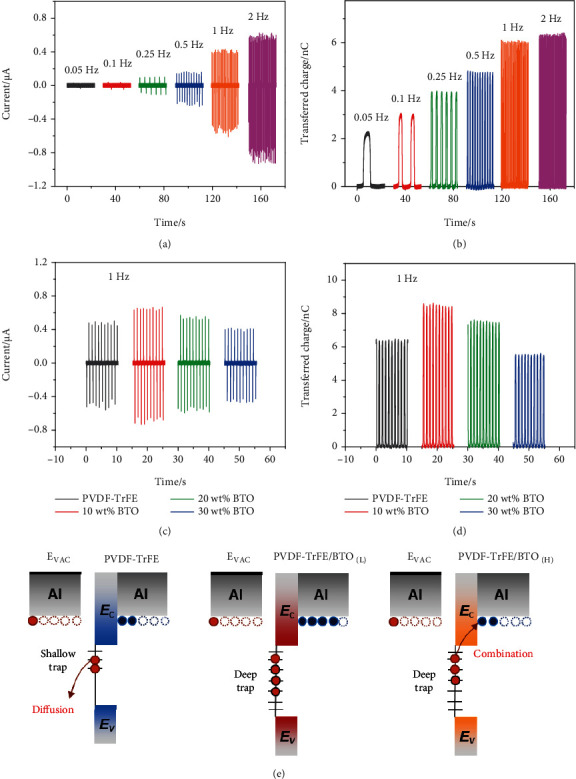
(a) Output current and (b) transferred charge of PVDF-TrFE-based triboelectric device under different stimulated frequencies. (c) Output current and (d) transferred charge of BTO/PVDF-TrFE-based triboelectric devices under 1 Hz. (e) Schematic working mechanism in BTO/PVDF-TrFE-based triboelectric devices.

The characterization of triboelectric devices assembled by BTO/PVDF-TrFE nanocomposites is depicted in Figures 5(a)–5(d). To verify the charge dissipation on the dielectric surface, PVDF-TrFE-based device was investigated under different frequency. As can be seen in Figure 5(a), the output current of the device increases with operating frequency in the range of 0.05 Hz-2 Hz. The increased current has been attributed to the result of fast charge transfer, where the charge dissipation was not considered [61, 62]. In Figure 5(b), one can see that more charges were transferred at a higher frequency. Based on the above analysis, it can be easily understood that the device operating at a lower frequency could suffer a longer period for charge dissipation at each cycle. Therefore, high output performance can be achieved for the device operating at a relatively high frequency, due to the reduced charge dissipation on the tribomaterial after contact electrification.

In Figures 5(c) and 5(d), the influence of BTO content on the device performance was investigated at a fixed frequency of 1 Hz. The output current and transferred charge both present a maximum value at a 10 wt% BTO content and then decrease with further BTO addition (similar as the voltage tendency in Figure S6). The working mechanism on different BTO/PVDF-TrFE nanocomposites are schematically compared in Figure 5(e). Specifically, as the surface potential of BTO/PVDF-TrFE nanocomposites is lower than that of Al foil (Figure S7), electrons are transferred from Al to tribomaterials and the transferred electrons (red dots) stored in the trap sites of the sample could induce positive charges (blue dots) on the back Al electrode. Due to the low capacitance and small number of trapping sites, electrons on PVDF-TrFE could diffusion and decrease the induction of positive charges on the bottom electrode, while, in the nanocomposites with high BTO content, the electrons could combine with induced positive charges via a large leakage current. Meanwhile, the exposed BTO NPs on the nanocomposite surface could significantly reduce the transferred electrons from Al friction layer (shown in Figure S8), as the electronegativity of BTO is lower than PVDF-TrFE, which has a tremendous charge-attracting and transfer properties [63].

**Figure 6:**
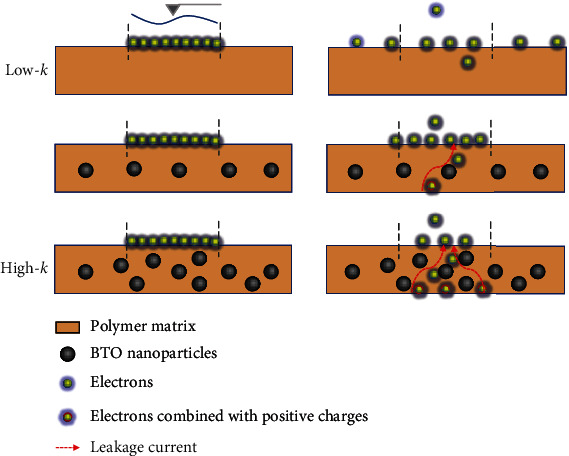
Schematic illustration of dielectric manipulated charge dynamic process under contact electrification.

Based on the above discussion, the dielectric manipulated charge dynamic behavior during contact electrification is proposed and demonstrated in Figure 6. Due to the different electron affinity between the tip/Al and the dielectric sample, electrons will transfer from the tip/Al to the sample during contact electrification, while, in the atmosphere, electrons can be combined with the opposite charges or hydrated ion from the atmosphere, and screened by water moisture. Meanwhile, charge diffusion and drift occurring on the sample can significantly reduce the surface charge density. PVDF-TrFE with low polarization and capacitance has a low ability to trap or sustain electrons, thus leading to a high charge diffusion rate, while the addition of high-*k* BTO NPs could efficiently improve the capacitance and electron trapping ability of the nanocomposites, as well as the inducibility of positive charges on the back electrode. Otherwise, the nanocomposites with BTO NPs also aggravate the electrons drifting to the back electrode, thus leading to an increase of leakage current and a significant decrease of surface charge density. Hence, in the view of charge generation, charge storage, and charge dissipation, it is essential to increase the electronegativity, capacitance, charge trapping ability, and suppress the leakage current of the tribomaterial for the construction of high-performance triboelectric devices.

## 3. Conclusions

In summary, an in situ method was demonstrated to characterize the contact electrification and charge dynamics of a metal-dielectric case at nanoscale via the combination of AFM and KPFM. Combined with the characterization of triboelectric device at macroscale, the influence of contact force, friction cycle, and dielectric properties on the contact electrification and charge dynamics were systematically investigated. Results indicate that the contact force is directly proportional to the transferred electrons from the tip/Al to PVDF-TrFE, and the electron density increases and tends to reach saturation with the increasing friction cycles. Due to the low capacitance of PVDF-TrFE, the transferred electrons rapidly diffuse to the adjacent areas, leading to a drastic decrease of electron density at the rubbed area. Significantly, the addition of high-*k* BTO NPs into a low-*k* polymer matrix can increase the capacitance and charge trapping capability of the corresponding nanocomposite, leading to an efficient inhibition of electron diffusion on the BTO/PVDF-TrFE nanocomposites and an enhancement of output performance of the triboelectric device. However, excessive BTO NPs could intensify the vertical drift of electrons into the bulk and the combination of drifting electrons with induced positive charges on the back electrode, which form a large leakage current, accelerate the dissipation of electrons, and degrade the device performance. Hence, BTO NPs play a dual role in the charge dynamics of the nanocomposites. Overall, this research elucidates the mechanism of dielectric manipulated charge transfer, charge distribution, charge dissipation, and provides perspectives for constructing high-performance triboelectric devices in the future.

## 4. Experimental Section

### 4.1. Fabrication of the BTO/PVDF-TrFE Nanocomposite Films

The complex BTO/PVDF-TrFE solution was prepared by adding 1 g BTO NPs (average diameter: 200 nm; Shandong Sinocera Functional Materials Co., Ltd., China; characterized in Figure S9) and PVDF-TrFE (molar ratio 8 : 2) into 6 ml *N*,*N*-dimethylformamide (DMF; analytical grade; Tansoole (China)). Firstly, BTO were ultrasonically dispersed in DMF for 30 min; then, PVDF-TrFE was dissolved in DMF by stirring for 24 h. The fraction of BTO was prepared at a certain fraction (0-30 wt%) according to the total mass of BTO and PVDF-TrFE. The complex solutions were spin-coated on heavily doped Si substrates (electrical resistivity < 0.0015 *Ω*∙cm; Shunsheng Electronic Technology Co., Ltd., China) at various rotation speeds to achieve a similar film thickness of 1.17 ± 0.1 *μ*m (characterized in Figure S10). The surface roughness *R*_q_ of the films increases from 9.27 nm to 50.7 nm with the increasement of BTO content in PVDF-TrFE (characterized in Figure S11). In addition, the BTO/PVDF-TrFE films used for constructing the triboelectric devices were fabricated by bar coating with a thickness of 18 ± 1.5 *μ*m. Subsequently, the nanocomposite films were dried at 60°C for 1 h and then annealed at 140°C for 2 h.

### 4.2. Construction of the Triboelectric Devices

The BTO/PVDF-TrFE nanocomposite films were tailored and attached with an Al foil back electrode (2.5 cm × 2.5 cm), and the friction counterpart was the bare Al foil.

### 4.3. Characterization and Measurements

The surface morphology of the nanocomposite films was characterized by SEM (Nova NanoSEM 450, FEI, USA). XRD (D/max-2200/PC, Rigaku, Japan) with Cu K*α* source was applied to investigate the crystalline structure of the films. The dielectric constant and loss of the nanocomposites were measured by a Novocontrol Alpha-N high-resolution dielectric analyzer (GmbH Concept 40) in the frequency range of 10^2^-10^6^ Hz at room temperature. Polarization-electric field (P-E) loop and leakage current were performed via a Precision Multiferroic Material Analyzer equipped with a Precision 10 kV HVI-SC and Trek MODEL 609B (Radiant Inc.). The isothermal surface potential decay experiments were conducted by corona charging the samples for 10 min under a needle-grid electrode system with the needle and grid voltages of -8 and -2 kV, respectively. The surface potential distributions were recorded with a high-speed electrostatic voltmeter (Trek, Model P0865). All experiments are carried out in an airtight chamber with the temperature and relative humidity keeping constant as ~27°C and~52%. Surface potential and the in situ contact electrification were investigated using an atomic force microscope (AFM, Dimension Icon, Bruker, USA) system under KPFM mode and contact mode, respectively. The measurements were performed under the same ambient conditions for all samples (temperature~25°C, humidity ~50%). Pt-coated Si probe (AC240TM) was produced by OLYMPUS. In contact mode, the normal contact force was calculated from the product of inverse optical lever amplification (112 nm/V), deflection setpoint (0-0.7 V), and spring constant of the tip (2 N/m). The corresponding contact forces related to the setpoints of 0 V, 0.1 V, 0.3 V, 0.5 V, and 0.7 V are 0 nN, 22.4 nN, 67.2 nN, 112 nN, and 156.8 nN, respectively. In KPFM mode, the tapping amplitude was set to be 350 mV, the lift height was 50 nm, and the scan rate was 2 Hz. Film thickness was measured using a profilometer (Alpha-step D-600, USA). The short current of the triboelectric devices mounted on a linear motor was recorded using a source meter (2450, Keithley).

## Data Availability

The data that support the findings of this study are available from the corresponding author upon reasonable request.

## Supplementary Materials

Supplementary Materials Fig. S1: derived peak *Δ*CPD as a function of deflection setpoint (a) and number of friction cycle (b). Fig. S2: dielectric loss of PVDF-TrFE and BTO/PVDF-TrFE nanocomposites. Fig. S3: charge induction on the back electrode for the samples with different electric polarization ability. Fig. S4: the *Δ*CPD distribution, cross-section profile, and the corresponding distribution width of 40 wt% BTO/PVDF-TrFE nanocomposite measured at 0 min. Fig. S5: distribution width of the Gaussian function fitting with the *Δ*CPD profiles of PVDF-TrFE and BTO/PVDF-TrFE nanocomposites measured at 0-12 min. Fig. S6: output voltage of BTO/PVDF-TrFE-based triboelectric devices under 1 Hz, 20 N. Fig. S7: average surface potential of (a) Al foil and (b) BTO/PVDF-TrFE nanocomposites. Fig. S8: (a) surface morphology of the BTO/PVDF-TrFE nanocomposites. (b) Local surface morphology and potential of the BTO/PVDF-TrFE nanocomposite. (c) The potential distribution along the dotted-line in (b). Fig. S9: SEM image and size distribution of BTO nanoparticles. Fig. S10: the thickness of the fabricated sample films. Fig. S11: the surface roughness of the fabricated sample films.
