# Supplementary material for: Dielectric Manipulated Charge Dynamics in Contact Electrification
Source: Research (Wash D C). 2022 Feb 1;2022:9862980. doi: 10.34133/2022/9862980 (PMC8829537; doi:10.34133/2022/9862980)
Supplement: Supplementary Materials — Fig. S1: derived peak ΔCPD as a function of deflection setpoint (a) and number of friction cycle (b). Fig. S2: dielectric loss of PVDF-TrFE and BTO/PVDF-TrFE nanocomposites. Fig. S3: charge induction on the back electrode for the samples with different electric polarization ability. Fig. S4: the ΔCPD distribution, cross-section profile, and the corresponding distribution width of 40 wt% BTO/PVDF-TrFE nanocomposite measured at 0 min. Fig. S5: distribution width of the Gaussian function fitting with the ΔCPD profiles of PVDF-TrFE and BTO/PVDF-TrFE nanocomposites measured at 0-12 min. Fig. S6: output voltage of BTO/PVDF-TrFE-based triboelectric devices under 1 Hz, 20 N. Fig. S7: average surface potential of (a) Al foil and (b) BTO/PVDF-TrFE nanocomposites. Fig. S8: (a) surface morphology of the BTO/PVDF-TrFE nanocomposites. (b) Local surface morphology and potential of the BTO/PVDF-TrFE nanocomposite. (c) The potential distribution along the dotted-line in (b). Fig. S9: SEM image and size distribution of BTO nanoparticles. Fig. S10: the thickness of the fabricated sample films. Fig. S11: the surface roughness of the fabricated sample films. [file 9862980.f1.docx]

**Electronic Supplementary Information**

**Dielectric Manipulated Charge Dynamics in Contact Electrification**

**Kunming Shi^a^, Bin Chai^a^, Haiyang Zou^b^, Daomin Min^c^, Shengtao Li^c^, Pingkai Jiang^a^, Xingyi Huang^a,^***

^a^Department of Polymer Science and Engineering, Shanghai Key Laboratory of Electrical Insulation and Thermal Aging, State Key Laboratory of Metal Matrix Composites, Shanghai Jiao Tong University, Shanghai 200240, PR China

^b^School of Materials Science and Engineering, Georgia Institute of Technology, Atlanta, GA 30332-0245, USA

^c^State Key Laboratory of Electrical Insulation and Power Equipment, Xi’an Jiaotong University, Xi’an, Shaanxi, 710049, PR China

*To whom correspondence should be addressed. E-mail: [xyhuang@sjtu.edu.cn](mailto:xyhuang@sjtu.edu.cn) (X.Y. Huang)


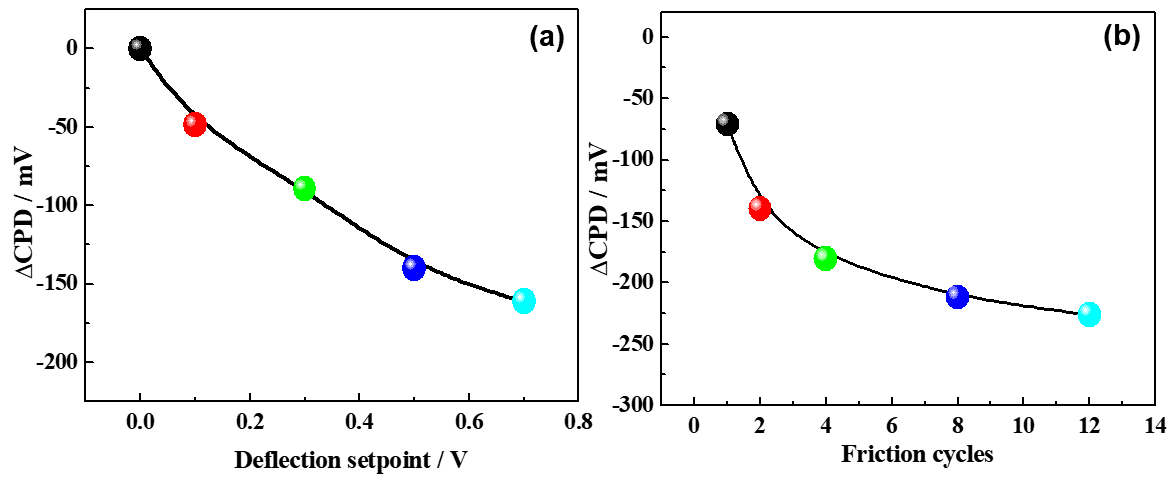


**Fig. S1** Derived peak ΔCPD as a function of deflection setpoint (a) and number of friction cycle (b).

**Fig. S2** Dielectric loss of PVDF-TrFE and BTO/PVDF-TrFE nanocomposites.


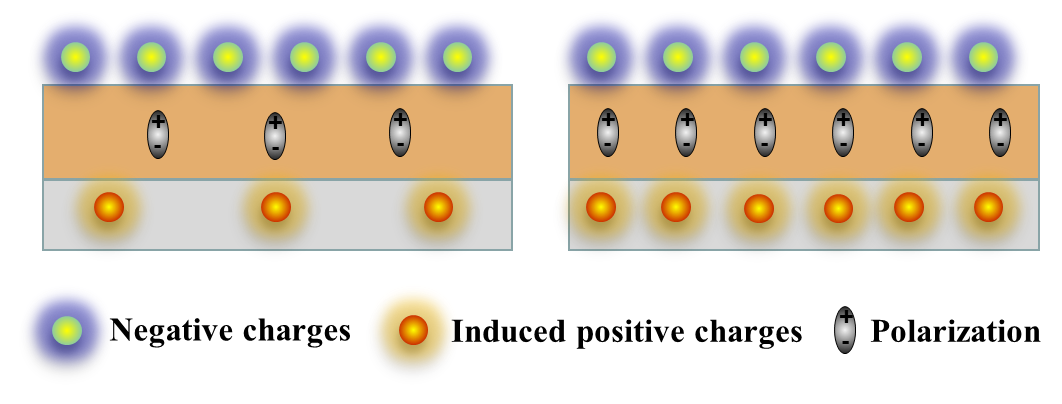


**Fig. S3** Charge induction on the back electrode for the samples with different electric polarization ability.


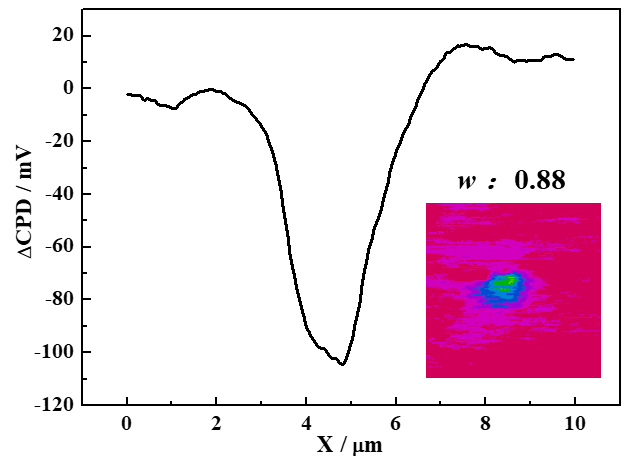


**Fig. S4** The ΔCPD distribution, cross-section profile and the corresponding distribution width of 40 wt% BTO/PVDF-TrFE nanocomposite measured at 0 min.

**Fig. S5** Distribution width of the Gaussian function fitting with the ΔCPD profiles of PVDF-TrFE and BTO/PVDF-TrFE nanocomposites measured at 0-12 min.

Figure S6. Output voltage of BTO/PVDF-TrFE based triboelectric devices under 1 Hz, 20N.


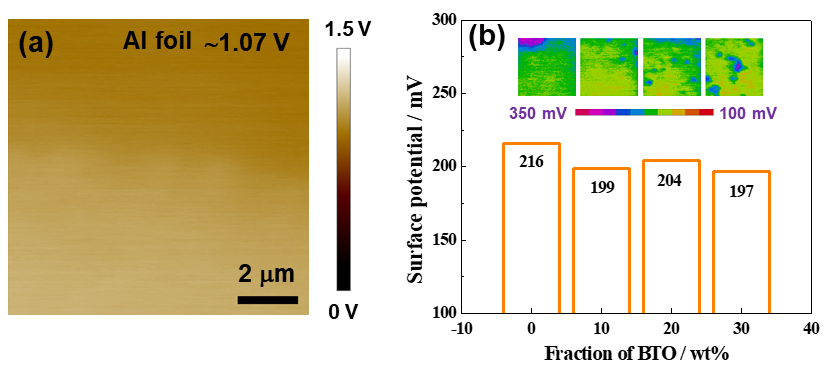


**Fig. S7** Average surface potential of (a) Al foil and (b) BTO/PVDF-TrFE nanocomposites.


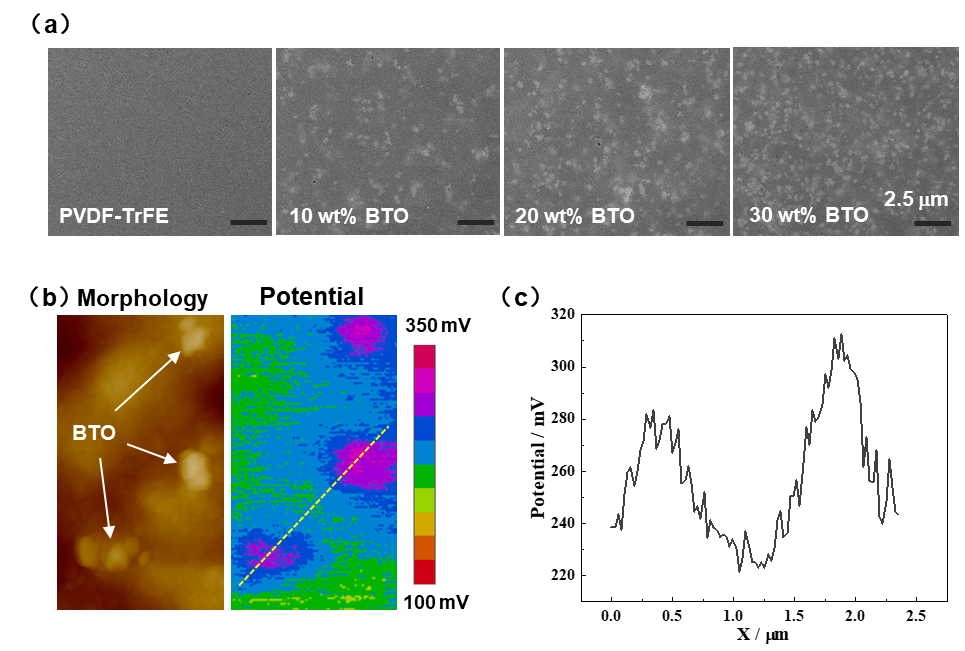


**Fig. S8** (a) Surface morphology of the BTO/PVDF-TrFE nanocomposites. (b) Local surface morphology and potential of the BTO/PVDF-TrFE nanocomposite; (c) The potential distribution along the dotted-line in (b).


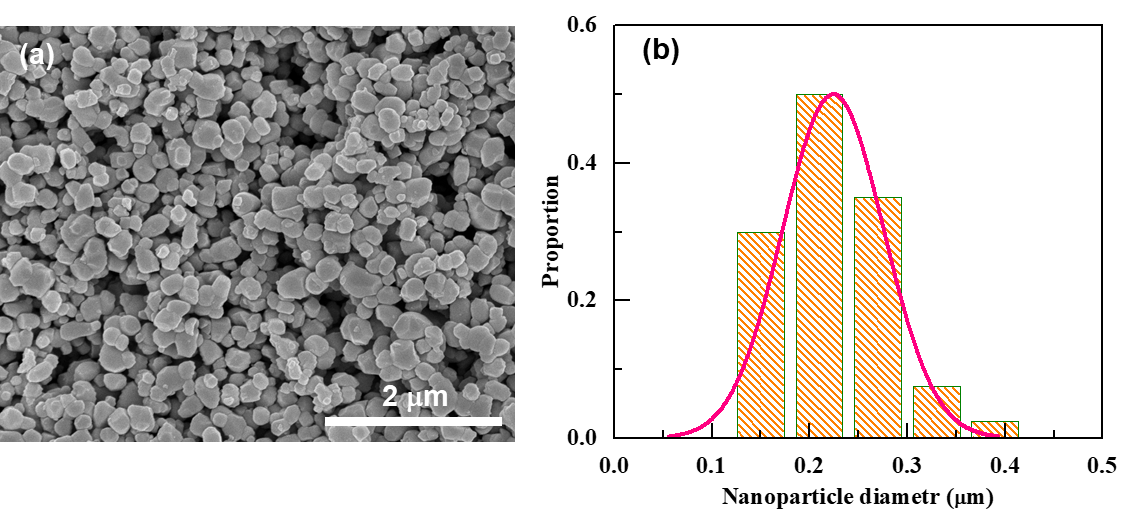


**Fig. S9** SEM image and size distribution of BTO nanoparticles.

**Fig. S10** The thickness of the fabricated sample films.


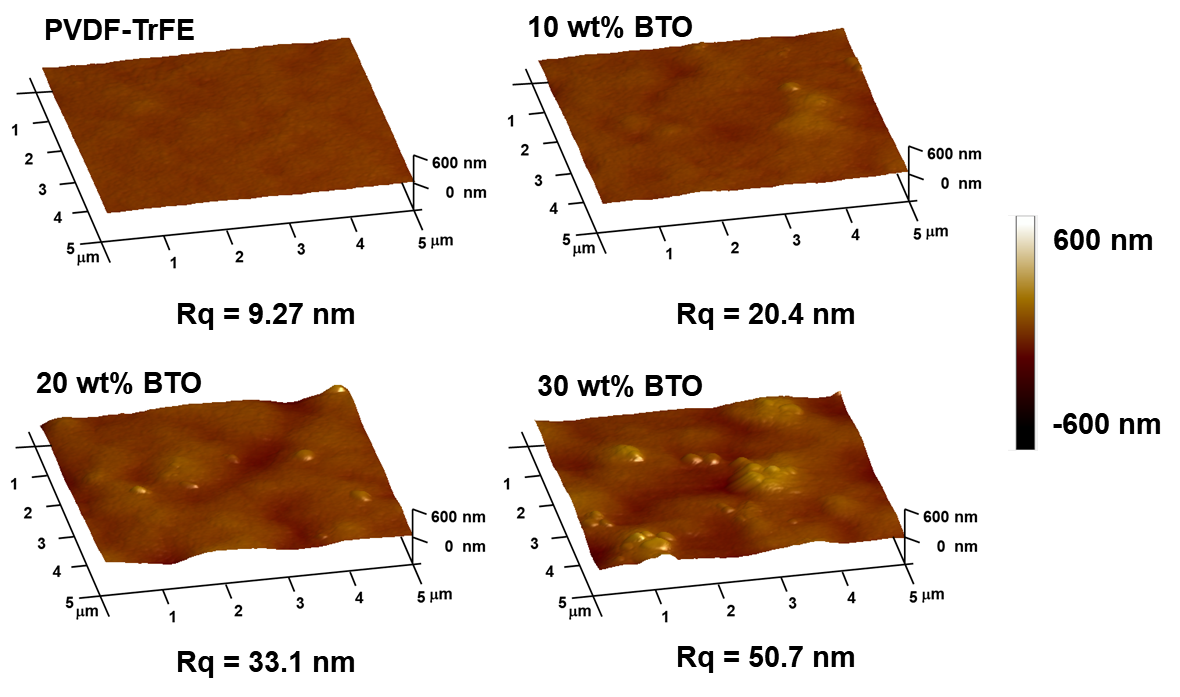


**Fig. S11** The surface roughness of the fabricated sample films.
